# Supplementary material for: Construction of a Form for Users of the Child Welfare System Based on the Delphi Method
Source: Children (Basel). 2023 Jun 7;10(6):1026. doi: 10.3390/children10061026 (PMC10297239; doi:10.3390/children10061026)
Supplement: Supplementary file 1 [file children-10-01026-s001.zip › children-2414003-supplementary.pdf]

Child and Adolescent Welfare System Form (CAWSys)

(Fernández-García, Gil-Llario, & Ballester-Arnal, 2023)

A. GENERAL INFORMATION

A.1. **Sex assigned at birth:**
- ☐ Woman
- ☐ Man

A.2. **Sexual orientation**:
- ☐ Heterosexual
- ☐ Homosexual
- ☐ Bisexual
- ☐ I am not sure

A.3. **Date of birth: ____________________**

A.4. **Nationality: ______________________**

A.5. **Unaccompanied migrant child:**
- ☐ Yes
- ☐ No

A.5.1 If yes, please specify when he/she arrived in Spain: ________ (month)

A.6. **Disability**:
- ☐ Yes
- ☐ No

A.7. **Physical health problems:**
- ☐ Yes
- ☐ No

7.1 If yes, please specify: ______________________

A.8. **Mental health problems:**
- ☐ Yes
- ☐ No

A.8.1 If yes, please specify: ______________________

A.8.2 Receives external treatment:
- ☐ Yes
- ☐ No

A.9. Consumption of psychoactive substances:

 Yes

A.9.1 Alcohol

 Sporadically
 Frequently
 No

A.9.2 Drugs

 Sporadically
 Frequently
 No

 No

B. SCHOOL/WORK SITUATION

B.1. Current school/work situation:

 Studies

 Primary education (indicate school year: ______)
 Secondary education (indicate school year: ______)
 Vocational Education and Training (indicate school year: ______)
 Certificate of Higher Education (indicate school year: ______)
 General Certificate of Education (indicate school year: ______)
 Special Education (indicate school year: ______)
 Non-regulated course (indicate which one: ______)

 Works
 None of the above

B.2. Has any school adaptation?

 Yes (indicate which one: ________)
 No
 N/A

B.3. Integration in the school:

 Good
 Regular
 Bad
 N/A

B.4. Behaviour in the classroom:

 Positive
 Negative
 N/A

B.5. **Has been expelled from the school?**

 Yes (no. of times____)
 No
 N/A

B.6. **Attitude and motivation towards learning:**

 Low
 Moderated
 High
 N/A

B.7. **School habits/skills:**

 Low
 Moderated
 High
 N/A

B.8. **Has repeated a grade?**

 Yes
 No
 N/A

B.9. **Truancy**:

 Yes
 No
 N/A

C. CHILD WELFARE SYSTEM HISTORY

C.1. **Age of entry into the child welfare system: ______** years

C.2. **Current legal status of the minor person:**

 Guardianship
 Custody

C.3. **Event giving rise to the placement:**

 Homeless situation
 Application for Voluntary Guardianship by parents or guardians
 Formalisation of a situation of fact of foster care

C.4. **Years in the child welfare system: ______** months

C.5. **Past protection measures:**

 Yes
 No

C.5.1 Which one: ____________________________

C.5.2 Duration of each one: _______ (months)

C.5.3 Reason for termination:

- At the request of the child/adolescent
- At the request of the family
- At the request of the administration
- For family reintegration
- As foreseen

C.6. Current protection measure:

- Foster care
- Residential care
  - Reception residence / home
  - Home / Residence for behavioural problems
  - General care residence / home
  - Home/Residence migration project
  - Emancipation home

C.6.1 Months enjoying the measure: ____________

C.6.2 Degree of adaptation/satisfaction with the measure:

- None
- Low
- Moderated
- Quite
- Total

C.6.3 Final aim of the intervention:

- Family reunification
- Definitive incorporation into a new family nucleus
- Preparing for emancipation
- Referral to a resource specific to their needs
- Not yet known

D. FAMILY VISITATION HISTORY

D.1. Are there established visits?

- Yes
- No

D.2. **Are they occurring?**

☐ Yes
☐ No

D.3. **Place of the visits:**

☐ Open
☐ Close

D.4. **Frequency of the visits:**

☐ Weekly
☐ Fortnightly
☐ Monthly
☐ No periodicity

D.5. **Duration of the visits:**

☐ Weekend
☐ Full day
☐ More than 4 hours (not full day)
☐ Less than 4 hours

D.6. **Control of the visits:**

☐ Yes
☐ No

D.7. **Persons with whom the child/adolescent is seen:**

☐ Parents
  ☐ both ☐ only mother ☐ only father
☐ Siblings
☐ Maternal grandparents
☐ Paternal grandparents

D.8. **Compliance with visits:**

☐ Never
☐ Rarely
☐ Quite often
☐ Always

D.9. **Assessment of visits by the minor person (in general)**:

☐ At odds
☐ Neutral
☐ Agree
☐ Always

E. BIOLOGICAL FAMILY INFORMATION

E.1. Filio-parental violence:

- ☐ Yes
  - ☐ Father
  - ☐ Mother
- ☐ No
- ☐ N/A

E.2. Mother

E.2.1 Background in the child welfare system:

- ☐ Yes
- ☐ No
- ☐ N/A

E.2.2 Physical health problems:

- ☐ Yes (specify: ________________)
- ☐ No
- ☐ N/A

E.2.3 Mental health problems:

- ☐ Yes (specify: ___________)
- ☐ No
- ☐ N/A

E.2.4 Recognised degree of physical disability:

- ☐ Yes
- ☐ No
- ☐ N/A

E.2.5 Recognised degree of mental disability:

- ☐ Yes
- ☐ No
- ☐ N/A

E.2.6 Substance abuse:

- ☐ Yes (specify: ___________)
- ☐ No
- ☐ N/A

E.2.7 Victim of maltreatment:

- ☐ Yes
- ☐ No
- ☐ N/A

E.2.8 Criminal record:
- ☐ Yes
- ☐ No
- ☐ N/A

E.2.9 Employment status:
- ☐ Employed
- ☐ Unemployed
- ☐ Working without a contract
- ☐ Retired
- ☐ Compensation
- ☐ N/A

E.3. Father

E.3.1 Background in the child welfare system:
- ☐ Yes
- ☐ No
- ☐ N/A

E.3.2 Physical health problems:
- ☐ Yes (specify: ________________)
- ☐ No
- ☐ N/A

E.3.3 Mental health problems:
- ☐ Yes (specify: ___________)
- ☐ No
- ☐ N/A

E.3.4 Recognised degree of physical disability:
- ☐ Yes
- ☐ No
- ☐ N/A

E.3.5 Recognised degree of mental disability:
- ☐ Yes
- ☐ No
- ☐ N/A

E.3.6 Substance abuse:
- ☐ Yes (specify: ___________)
- ☐ No
- ☐ N/A

E.3.7 Victim of maltreatment:

- Yes
- No
- N/A

E.3.8 Criminal record:

- Yes
- No
- N/A

E.3.9 Employment status:

- Employed
- Unemployed
- Working without a contract
- Retired
- Compensation
- N/A

E.4. Siblings

- Yes

E.4.1 Number of siblings: ______

E.4.2 Place among the siblings: _____

E.4.3 Place where his/her siblings live:

- Live with him/her in the same residential care facility
- Live in another residential care facility
- Live with his/her biological family which is the same as the family of the child/adolescent concerned
- Lives with his/her biological family that is different from that of the minor person in question
- Lives with other family members
- Lives with a foster family

- No

E.5. Economic situation

- High
- Medium
- Low
- Homeless
- N/A

E.6. Community environment

E.6.1 Conflicting social dynamics
- ☐ Yes (specify: __________)
- ☐ No
- ☐ N/A

E.6.2 Presence of a support network
- ☐ Yes (who: _______________)
- ☐ No
- ☐ N/A

F. EXPERIENCES OF SEXUAL ABUSE

F.1. Suspected sexual abuse:
- ☐ Yes
- ☐ No
- ☐ N/A

F.2. Confirmation of suspected sexual abuse:
- ☐ Yes
- ☐ No

F.3. Alleged perpetrator:
- ☐ Known (indicates who: _______________________)
- ☐ Unknown

F.4. Occasions on which it has occurred (approx.): _________________

F.5. Sex of alleged perpetrator:
- ☐ Male
- ☐ Female

F.6. Short and/or long-term consequences: ____________________________

F.7. Has received subsequent therapeutic support?
- ☐ Yes
- ☐ No
